# Withdrawing attempt and its related factors among Iranian substance users: a case-control study

**DOI:** 10.1186/s13011-018-0184-z

**Published:** 2018-12-06

**Authors:** Gholamhossein Shahraki, Zahra Sedaghat, Mohammad Fararouei

**Affiliations:** 1 Social Determinants of Health Research Center, Yasuj University of Medical Sciences, Yasuj, Iran; 2 Research Center for Health Sciences, Department Epidemiology, Shiraz University of Medical Sciences, Shiraz, Iran; 3 HIV/AIDs Research Center, Shiraz University of Medical Sciences, Zand street, Shiraz, 7143854188 Iran

**Keywords:** Substance use, Current drug users, Withdrawing substance dependence

## Abstract

**Background:**

Substance dependence is considered as an important health disorder with a wide and serious range of psychosocial effects. With regard to the large number of people with substance dependency in Iran and high failure rate of quitting attempts, the aim of this study was to identify contributing factors to quitting substance dependency among patients in Yasuj the capital of Kohgilooyeh and Boyerahmad province.

**Methods:**

This case-control study was conducted on 150 current substance users (as control group) and 187 patients who voluntarily introduced themselves to governmental and private residential treatment camps (as case group). The participants in the case group were selected via two stage cluster sampling among those admitted to residential treatment camps. Those in the control group were selected via snowball sampling among current substance users.

**Results:**

Based on the results from multiple logistic regression analysis, significant associations were observed between attempting to withdraw substance use and father’s education (OR _high school or university /illiterate_ = 0.36, 95%CI: 0.18 to 0.72, *P* = 0.004) and smoking (OR _yes/no_ = 4.26, 95%CI = 1.90 to 9.57, *P* < 0.001) were identified.

**Conclusions:**

This study introduced father’s education as an obstacle to attempting to quit substance dependency. This finding can be justified by easier access to money and therefore less financial pressure in providing drugs among those with wealthier families. Also, smoking was more common among those who registered with the camps. This is possibly due to the quitters attempt to replace the drugs with cigar smoking as an alternative. Training families in helping their members in preventing or quitting substance dependency is a potentially useful approach. Studies are needed to define whether the common belief that smoking helps in withdrawing substance use is helpful.

## Background

Substance dependence (SD) is considered as an important health and psychological disorder with wide and serious psychosocial effects. Substance dependence is also an important contributor to both family and economic harms [1]. Along with the social and demographic changes, SD is becoming a more serious health and social issue as the number of substance users is rising constantly among both adults and young individuals [2, 3]. Substance dependence is associated with a wide range of side effects such as sexually and blood transmitted diseases, depression and suicide, problems in interpersonal relationships and traffic related injuries or deaths [4, 5]. Depending on the age of starting substance use, the disorder also causes several cognitive, social, behavioral and other health problems [3]. It is suggested that adverse effects of SD among young individuals are even more serious as over 90% of substance users who obtain their first experience of administering the substances during their adolescence, experience serious adverse health and psychological effects and socio-economic problems [4, 6, 7]. Based on a report from the united nations office on drug (UNODC), there are approximately 185 million substance users worldwide, 5% of whom (nearly 20 million) used substances for at least 1 year [2, 8–10]. It is estimated that 1.2 to 2% of drug users are living in Iran [3, 11]. More worryingly, possibly due to its geographical location, Iran is experiencing a sharp raise of SD [2, 3]. It is also worth noticing that SD among Iranian men is about ten times more common than women. It is possibly due to men’s easier social, cultural and financial access to the substances via attached social networks. Official reports suggest that opium, cocaine and heroin are the most common types of illicit drugs used in the country [12]. In addition to opioids, chemosynthetic substances including oxy Sudan, hydro soudan, methadone, hydromorphone, meperidine and codeine are also widely used in Iran. Opioids are powerful pain killers which join to both central and environmental receptors [13] and cause some cognitive and socio-behavioral alterations including social disability, depression and anxiety among users [13, 14]. In addition to the individual affected side effects, drug trade activities including drug trafficking also has caused security and social crisis in Iran [3].

As the result of adverse health, social and financial effects of substance dependence in Iran, the issue of SD epidemic is of grate national concern [3, 8]. Due to variability and complexity of SD, the contributing factors are largely different between communities [3]. In addition, despite the ease of becoming SD to different available substances, especially opioids, withdrawal is hard, complicated, lengthy and painful with a worryingly high rate of failure even after several years of staying clear of drugs. Although the painful symptoms among people in treatment for substance use disorder start few hours after the last usage of drug, they may last for about 1 month, after which the symptoms start weakening [15]. Numerous studies suggested that depending on the type of used substances and method of administration, 20 to 90% of substance users use treatment and quitting procedures, though predominantly with no success [3]. For example, a survey on a representative sample of Iranian population suggested that about 40% of opioids dependent individuals who were predominantly male, had 12 month of unmet treatment need [16]. Among different methods of withdrawing SD, using alternative drugs including bupropion, medafinil, dextoam and phetamine is very common [17]. Two other substances which are wildly used for treatment of SD among Iranian patients are methadone and methamphetamine. Previous studies suggested a sharp increase (6 to 20%) in the use of methamphetamine and methadone among Iranian withdrawers as alternative to opioids [17]. However, solely treatment with methadone or methamphetamine is Longley and the drugs bring dependency too. This make treatment of SD even more complicated [18]. Registering with residential rehabilitation centers is also among popular methods used by Iranian substance users to quit. Rehabilitation camps help patients in changing their social environment, provide them with better care and make them feel safer [14]. These centers also play important roles in changing patient’s behavior and social networks which help them to increase the chance of successful treatment [14]. It seems that many Iranian drug users try to stop substance use several times with different self-administered methods including remedies and treatment approaches. However, due to involvement of many psycho-social, financial and family factors in SD, most quitting attempts are failed and the patient starts substance use again [19]. The patients then may try more advanced methods including registering in residential treatment camps. Few studies are conducted to investigate the related factors of quitting SD. For example, a study on characteristics of Iranian substance users who voluntarily introduced themselves to residual treatment centers suggested that most withdrawers were married, were literate and were employed [19]. According to the results, most participants were trying to quit SD because of physical, psychological or financial problems. It was interesting that family encouragement and support was only the fourth reported reasons for quitting SD. Regarding the high prevalence of substance use and the sharp increase of SD [3] prevention and treatment of SD has become a strategic priority of Iranian government. The aim of this study was to identify factors which contribute to attempt to treat substance use disorder among males users in Yasuj the capital of Kohgilooyeh and Boyerahmad province.

## Methods

This case-control study recruited a sample of male participants aged 18 to 45 (no matching) who were current drug users (controls, *n* = 142) or those who were voluntarily admitted to residential treatment camps (cases, *n* = 187). More details about the methods of sampling and data collection among controls are presented before via reporting the results of the first phase of the study [20]. Also, to reach the required sample size for the case group (withdrawers), in addition to those who were in the residential treatment camps at the time of snowball sampling, an independent sample was taken from those who were admitted to substance dependency treatment centers.

### Selection of cases and controls

Both cases and controls are restricted to male participants. Due to no or hard access to those who were substance users in the community (substance use is a serious crime in Iran), the participants in the control group were selected via snowball sampling with the help of several substance user volunteers, who were themselves substance users, in finding individuals with substance use disorder using drugs on a daily bases to deliver the questionnaire and collect it when completed. Those attempting to treat substance use (the case group) were selected via an independent systematic random sampling from those who were under treatment in rehabilitation camps or other substance dependency treatment centers at the time of study. Due to governmental subsidiaries there was no or small registration fees and treatment costs for the voluntaries. Moreover, the selected medicine (e.g. methadone) is only provided by the government to those who registered with the centers. It is therefore believed that the registered subjects were a representative sample of the withdrawing drug users as no other competitive way was known to be available to the drug users. The governmental and private treatment centers accept only those who willingly want to stop drug use as there is no facility to enforce substance users to follow the treatment procedures. The study was conducted in Yasuj (the capital of Kohgilooyeh and Boyerahmad province located at southwestern part of Iran). In this study, drug dependence was defined as “compulsive and repetitive drug administration”. Drug dependence among current drug users and those under treatment was self-reported. This was done due to the fact that there was no direct access to the controls (current drug users).

### Data collection

A self-administered questionnaire (details about constructing and validating procedures are provided before) [20] was used to collect required information on the participant’s demographic, social and behavioral characteristics. The questionnaire was designed to collect information on the participant’s educational, social and behavioral characteristics as well as the history of their substance dependence and related behaviors.

The substance dependent individuals who were found by the substance user research assistants, were directed to a private place in which the questionnaire was delivered. No name or identifying information was required. To recruit case group, those who registered to rehabilitation camps or any other private or public treatment centers and were not using drugs at the time of interview were asked to complete the questionnaire in a private place. Similar to the case group, no name was required.

Study variables: The questionnaire consisted of two sections; a section with a wide range of information which included: demographic (birth order, number of sisters and brothers, marital status, job, level of education), family (father’s and mother’s education and job, living with spouse if married, lived with family when was single, substance use among relatives of the participant) and behavior (smoking, alcohol consumption, criminal history).

### Inclusion criteria

Participants were all male and had minimum level of education (to be able to read and answer the questions). Both case and control groups were healthy enough to read and fill the questionnaire.

## Sample size and statistical analysis

Sample size was calculated in order to detect an increase in the chance of attempting to quit as small as twice the baseline for those had compulsory or higher education. The alpha value and power were set at 0.05 (two sided) and 80% respectively. Bivariate analysis (using t and chi-square tests) was conducted as primary analysis strategy to determine unadjusted associations of the study variables with the chance of attempting to stop substance use during the period of study. To measure the adjusted associations of each independent variable with the chance of attempting to stop substance use, multiple logistic regression was applied. Variables were entered in to a logistic regression model using stepwise forward variable selection strategy. The modeling procedure was started after collinearity between the independent variables was measured using variance inflation factor index (VIF). The cut point for VIF was set to 10. SPSS version 19 is used for analysis of data.

## Results

Opioids (mostly opium) were the main substance used by the currently users (71.0%) and under treatment (75.8%) participants (chi-square = 0.92, df = 1, *P* = 0.34). Also no significant difference was found between currently users and under-treatment participants with regard to the duration of drug use (*t* = 1.11, df = 327, *P* = 0.14). The demographic and social characteristics of current substance users and withdrawers are compared and the results are presented in Table 1. Compared to the current users, withdrawers were more illiterate (11.54% among current users, 22.31% among withdrawers, chi-square = 14.47, df = 4, *p* = 0.006), were living with spouse if married (19.75% among current users compared to 34.58% among withdrawers, chi-square = 5.00, df = 1, *p* = 0.02) and were more likely to be smoker (72.86% among current users compared to 90.00% among withdrawers, chi-square = 16.03, df = 1, *p* < 0.001). In addition, compared to current users, family of withdrawers were more aware of their child’s (the participant’s) substance dependence (37.50% among drug users compared to 45.45% among those who were attempting to stop substance dependence, chi-square = 1.53, df = 1, *p* = 0.22). Interestingly, fathers of currently drug users had predominantly higher education (fathers of 65.14% of current users had elementary or university degrees compared with 45.03% of fathers among quitters, chi-square = 10.54, df = 2, *p* = 0.005). Baseline characteristics of participants suggested no significant difference in other demographic characteristics between the two groups.

**Table 1:** Baseline characteristics of participants

Variables	Current users (*n* = 142)	In treatment for substance use disorder (*n* = 187)	*p*-value*	Test value
Quantitative measures	Mean ± SD	Mean ± SD		
Age	34.49 ± 9.63	34.81 ± 9.00	0.76**	−0.29
Duration of drug usage (year)	9.77 ± 8.86	11.19 ± 6.83	0.93**	1.11
Age of starting cigarette (year)	17.82 ± 5.43	17.55 ± 4.68	0.67**	0.42
Amount of drug used (times per day)	2.53 ± 2.26	2.89 ± 3.15	0.34**	
Qualitative measures	*N*(%)	*N*(%)		
Marital statues				
Single	59 (42.14)	80 (43.72)	0.77	0.08
Married	81 (57.86)	103 (56.28)		
Number of sisters				
No sister	6 (4.58)	9 (5.52)	0.47	1.47
1	28 (21.37)	26 (15.95)		
≥2	97 (74.05)	128 (78.53)		
Number of brothers				
No brother	4 (2.99)	5 (2.96)	0.44	1.63
1	19 (14.18)	16 (9.47)		
≥2	111 (82.84)	148 (87.57)		
Education				
Illiterate/primary	29 (22.31)	21 (11.54)	0.006	14.47
Secondary	39 (30.00)	63 (34.62)		
High	6 (4.62)	22 (12.09)		
Diploma	34 (26.15)	58 (31.87)		
University	22 (16.92)	18 (9.89)		
Criminal history				
No	55 (55.00)	73 (51.77)	0.62	0.24
Yes	45 (45.00)	68 (48.23)		
Living with spouse (if married)				
No	65 (80.25)	70 (65.42)	0.02	5.00
Yes	16 (19.75)	37 (34.58)		
Dependence among relatives				
No	77 (62.60)	124 (75.15)	0.02	5.26
Yes	46 (37.40)	41 (24.85)		
Mother’s education				
Illiterate	20 (19.42)	33 (22.76)	0.77	0.51
Primary/ secondary	14 (13.59)	21 (14.48)		
High school/university	69 (66.99)	91 (62.76)		
Father’s education				
Illiterate	20 (18.35)	48 (31.79)	0.005	10.54
Primary/ secondary	18 (16.51)	35 (23.18)		
High school/university	71 (65.14)	68 (45.03)		
Mother’s job				
House holder	114 (94.21)	158 (96.93)	0.26	1.26
Employed	7 (5.79)	5 (3.07)		
Lived with family when was/is single				
Lived alone	41 (30.15)	46 (26.14)	0.43	0.61
Living with parents	95 (69.85)	130 (73.86)		
Do you Smoke				
No	38 (27.14)	18 (10.00)	< 0.001	16.03
Yes	102 (72.86)	162 (90.00)		
Family were aware of your dependence?				
No	60 (62.50)	84 (54.55)	0.21	1.53
Yes	36 (37.50)	70 (45.45)		

* Based on Chi-squire test; **Based on student t-test

The results of multiple logistic regression revealed that the level of father’s education and smoking are significant predictors of attempting to treat substance dependence. According to Table 2, significant inverse and direct associations between father’s education (OR _high school or university/illiterate_ = 0.36, 95%CI: 0.18 to 0.72, *P* = 0.004) and smoking (OR _yes/no_ = 4.26, 95%CI = 1.90 to 9.57, *P* < 0.001) with the chance of attempting to treat substance dependence were identified respectively.

**Table 2:** The adjusted associations of study variables with quitting attempt

Variable	OR	CI	*P*-value
Education			
Illiterate/primary	1.00	–	–
Secondary	1.37	0.56 to 3.31	0.48
High	3.26	0.89 to 11.92	0.07
Diploma	1.52	0.61 to 3.77	0.36
University	0.83	0.28 to 2.39	0.73
Father’s education			
Illiterate	1.00	–	–
Primary/ secondary	0.65	0.28 to 1.51	0.932
High school/university	0.36	0.18 to 0.72	0.004
Do you smoke			
No	1.00	–	–
Yes	4.26	1.90 to 9.57	< 0.001

## Discussion

This case-control study examined the relationship between a wide range of demographic, social and behavioral characteristics with withdrawing substance use several of which were introduced as contributing factors for drug use [20]. The results of bivariate analysis showed that there is no noticeable association between number of sisters or brothers and attempting to treat substance dependence. In other word, the presence or absence of siblings has no effect on withdrawal attempt. However, the results suggested that staying with spouse can positively encourage substance users to withdraw substance dependency. It seems that spouse can encourage and support her husband in deciding to treat substance dependence. In line with the results of current study, Van et al. reported that current substance users are not predominantly living with their families and their spouse. Indeed, it seems that leaving family is a contributing factor for continuing substance use [21]. This study also suggested that smoking is more common among those attempting to treat substance dependence compared to current users. This issue is not directly investigated by other researchers. However, results of a study in America suggested much higher smoking among substance users (comorbidity) compared to the American general population [22]. It seems that those who want to stop substance use try to replace the drug they use with cigar to reduce the severity of psychological and physiological withdrawal syndromes. This is possibly because they believe that cigar is less harmful than drugs.

The results of multivariate analysis suggested that higher level of education of substance user’s father is a preventing factor for stopping drug administration. Reports from several researchers suggested a direct and significant association between father’s education and his socio-economic status with children’s substance dependence [6, 23–25]. However, no published study has been found to report the effect of parents socioeconomic (particularly father’s education) status and child’s attempt to stop drug use. It is possible that children of parents with higher education have less problem in providing money for purchasing drugs and that being financially able to afford costs of providing drugs bring users under less pressure to stop substance use.

## Conclusion

This study introduced father’s education as an obstacle to withdraw SD among male substance users. This finding can be justified by the fact that in general families with more educated parents have expectedly better income compared to those with less educated parents. It is reasonable to assume that belonging to a wealthier family provides easier access to money and therefore less financial pressure in providing drugs among members of wealthier families. Smoking was more common among withdrawers. There are several possible explanations for this finding. For example, substance users may use cigar as a replacement to the substances to make withdraw easier. This is a generally accepted view among substance users and is supported by the camp managers by allowing the residences to smoke with no limits. Parents or spouse can take more roles in helping substance users to withdrawal. Moreover, the results suggested that family members especially sisters and brothers should have more effective roles in helping their substance addicted brothers to withdraw. Family of substance users should receive trainings and supports in helping their members not to become substance dependence at first place and second, to support those members who are substance users with treatment. High risk families should get these supports and helps by public or private providers.

## Limitations

This study used snowball sampling for selecting currently substance users. Inevitably, these methods of selection strategies may have caused selection bias. The study was merely based on the self-reported information provided by the participants and no attempt was made to contact parents or spouses as those with no or separated family members could not be included in the analysis causing differentiable missing data and selection bias. Although the results from Table 1 are mostly supported by the results from multiple logistic model, the results of the significant statistical tests presented in Table 1 may be affected by a bigger level of type I error due to the large number of tests. All data obtained from the participants and no contact was made to the family of the participants. As a result no data on the attitude of the parents to mental illness and addiction was available. No psychological assessment or test was conducted in order to save time due to the lower tempered participants during abstaining period in the camps.

## Abbreviations

SD: Substance dependence
UNODC: United nations office on drug
